# Editorial: Neurological dysfunction and diseases in high altitude

**DOI:** 10.3389/fneur.2023.1343786

**Published:** 2024-01-04

**Authors:** Xudong Wen, Pan Long

**Affiliations:** ^1^Department of Gastroenterology, Chengdu Integrated TCM & Western Medicine Hospital, Chengdu University of Traditional Chinese Medicine, Chengdu, Sichuan, China; ^2^Department of Ophthalmology, The General Hospital of Western Theater Command, Chengdu, Sichuan, China; ^3^School of Materials Science and Engineering, Southwest Jiaotong University, Chengdu, Sichuan, China

**Keywords:** neurological dysfunction, Editorial, high altitude, cerebral tissue oxygenation, visual working memory

High altitude attracts a large number of people, including travelers, athletes, military personnel, and businessmen. However, the high altitude environment is a serious challenge for these people who have been living at lower altitudes. Especially, neurological dysfunction, including cognitive function decline, memory deterioration, para-equilibrium, and somnipathy, is common symptoms for these people entering high altitude. Unfortunately, the mechanism, pathology, clinical presentations, effective treatments are not totally clear. Simplistically attribute those neurological dysfunctions to the hypoxia and hypothermia in high altitude is not appropriate. This Research Topic aims to provide a forum for researchers to discover the specific manifestations and related mechanisms of high altitude's short-, medium-, and long-term effects on neurological function and to explore the effective drugs, oxygen therapy, and exercise methods to improve neurological dysfunction at high altitude.

In this Research Topic, we have collected a total of six papers, which cover the latest hotspots of neurological dysfunctions, including cerebral tissue oxygenation and cerebrovascular reactivity with cross-sectional population-based study, brain-derived neurotrophic factor and visual working memory ability in high altitude. We thus have briefly introduced the papers published in the Research Topic as follows.

Previous studies primarily focused on high altitude cerebral edema, now more attentions have been paid to sleep disturbances and cognitive impairment at high altitudes. Zhang et al. systematically analyzed and visualized research on sleep disturbances and cognitive impairment at high altitudes using a bibliometrics method. They suggested that sleep disturbances and cognitive impairment at high altitudes would be a promising direction for high altitude neurological dysfunctions. Moreover, Yang et al. reviewed the potential roles of exosomes in hypoxic injury and adaptation. They conducted a comprehensive summary to assess the pathophysiological impact of exosomes on neurological dysfunctions under hypoxic conditions. Chen et al. summarized the performance, mechanism and prevention and treatment methods of hypobaric hypoxia environment affecting cognitive function, so as to provide theoretical reference for exploring and treating cognitive dysfunction at high altitude.

Bao et al. investigated the visual working memory ability of individuals at high altitude. They combined a lateral-change detection task with an event-related potential analysis to explore the changes in visual working memory capacity of individuals who migrated from low altitude to Tibet (high altitude). These results suggested that attentional resources that are chronically exposed at high altitude are more easily captured by task-irrelevant information. This may be due to impaired inhibitory control leading to their difficulty in excluding distractors from task-irrelevant information.

Luyken et al. evaluated the effects of long-term high-altitude residence on cerebral tissue oxygenation (CTO) and cerebrovascular reactivity in a prospective cohort study. PaO_2_ decreased and heart rate increased, but there was no significant change in CTO. Within 5 years, CTO was preserved despite a decrease in PaO_2_ in plateau residents. Since this is associated with a decrease in the responsiveness of cerebral blood volume to hypocapnia, an adaptation of cerebrovascular reactivity may have occurred.

Fan et al. investigated the relationship between brain-derived neurotrophic factor (BDNF), a member of the neurotrophic family, and attention of long-term high-altitude immigrants. They evaluated the relationship between peripheral blood concentrations of BDNF and the three attentional networks. Higher BDNF levels were associated with poorer executive control, suggesting that hypoxic brain damage may occur in individuals with relatively higher BDNF levels after long-term high-altitude exposure, and this higher BDNF level may be the result of self-repair in response to the adverse effects of the high-altitude environment.

While some progress has been made on the effects of high-altitude exposure on neurological dysfunctions and diseases, major problems on this topic remain largely unresolved. First of all, large-scale phenomics studies in high-altitude populations are needed to clarify the incidence, treatment and prognosis of neurological dysfunctions and diseases. Secondly, studies should focus on the morphological, functional and molecular biological changes of the brain and brain-periphery in the onset of high-altitude neurological dysfunctions and diseases. Thirdly, it is necessary to carry out more interventions for cognitive impairment at high altitude. Combined with artificial intelligence, polymer materials and other disciplines, dynamic markers should be developed for real-time detection. Small molecule drugs, traditional Chinese medicines, exercise therapy, oxygen therapy and other methods could be used to intervene.

## Author contributions

XW: Writing–original draft. PL: Writing–review & editing.

